# Epigenetic Methylation of Parathyroid *CaR* and *VDR* Promoters in Experimental Secondary Hyperparathyroidism

**DOI:** 10.1155/2012/123576

**Published:** 2012-10-10

**Authors:** Jacob Hofman-Bang, Eva Gravesen, Klaus Olgaard, Ewa Lewin

**Affiliations:** ^1^Nephrological Department P2132, Rigshospitalet, Herlev Hospital, University of Copenhagen, 9 Blegdamsvej, 2100 Copenhagen, Denmark; ^2^Nephrological Department B, Herlev Hospital, 9 Blegdamsvej, 2100 Copenhagen, Denmark

## Abstract

Secondary hyperparathyroidism (s-HPT) in uremia is characterized by decreased expression in the parathyroids of calcium sensing (*CaR*) and vitamin D receptors (*VDR*). Parathyroid hormone (PTH) is normalized despite low levels of *CaR* and *VDR* after experimental reversal of uremia. The expression of *CaR* in parathyroid cultures decreases rapidly. Methylation of promoter regions is often detected during epigenetic downregulation of gene expression. Therefore, using an experimental rat model, we examined changes in methylation levels of parathyroid *CaR *and *VDR* promoters *in vivo *and *in vitro*. *Methods*. Uremia was induced by 5/6 nephrectomy. Melting temperature profiling of *CaR* and *VDR* PCR products after bisulfite treatment of genomic DNA from rat parathyroids was performed. Real-time PCR measured expression of *PTH, CaR, VDR*, and *klotho* genes *in vitro*. *Results*. Parathyroids from uremic rats had similar low levels of methylation *in vivo* and *in vitro*. In culture, a significant downregulation of *CaR, VDR*, and *klotho* within two hours of incubation was observed, while housekeeping genes remained stable for 24 hours. *Conclusion*. In uremic s-HPT and *in vitro*, no overall changes in methylation levels in the promoter regions of parathyroid *CaR* and *VDR* genes were found. Thus, epigenetic methylation of these promoters does not explain decreased parathyroid expression of *CaR* and *VDR* genes in uremic s-HPT.

## 1. Introduction

Secondary hyperparathyroidism in uremia (s-HPT)—a disorder caused by progressive loss of kidney function, low levels of active vitamin D (1,25(OH)_2_D (calcitriol)), increased phosphate retention, and low levels of plasma ionized calcium (Ca^2+^) [1, 2]—results in the highly elevated synthesis and secretion of parathyroid hormone (PTH) and enlargement of the parathyroid glands in order to maintain normal plasma Ca^2+^ and phosphate levels.

The calcium-sensing receptor (CaR) plays a key role in maintaining of Ca^2+^ concentrations in extracellular fluids within a narrow range, primarily by modulating the function of the parathyroid glands. The CaR belongs to family C of the superfamily of seven transmembrane G-protein-coupled receptors. It regulates the biosynthesis and secretion of parathyroid hormone (PTH), as well as parathyroid cell proliferation, which is inhibited at high Ca^2+^ concentrations and stimulated at low Ca^2+^ concentrations. The effect of low calcium on PTH gene expression is posttranscriptional.

Another important regulator of the *PTH* gene, calcitriol, decreases *PTH* gene expression at the transcriptional level. Calcitriol's action is mediated via binding to the vitamin D receptor (VDR), a steroid hormone receptor. Once bound to calcitriol, the VDR forms a heterodimer with the retinoic X receptor and binds to the vitamin-D-responsive DNA element in the *PTH* gene promoter. VDR regulates the expression of many genes involved in mineral metabolism, cell proliferation, and differentiation.

**Figure 1:**
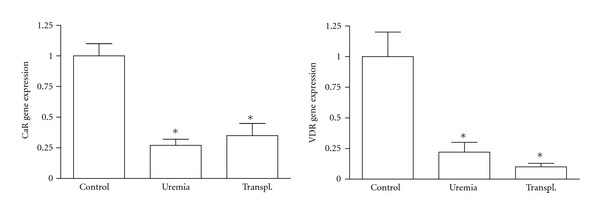
Downregulation of parathyroid *CaR* and *VDR* gene expression in uremia. The parathyroid calcium receptor (*CaR*) and vitamin D receptor (*VDR*) were significantly downregulated in long-term uremic rats with severe secondary hyperparathyroidism. *P < 0.005. Modified from [7], with permission.

In uremic patients, s-HPT may eventually turn into severe nodular hyperplasia, where the parathyroids cease to respond to Ca^2+^ and vitamin D therapy and continue to secrete significantly elevated amounts of PTH. This indicates a reduced presence or functional ability of CaR or VDR in these cells. Both clinical and experimental s-HPT are characterized by very low expression of the calcium-sensing receptor (*CaR*) and the vitamin D receptor (*VDR*) genes in the parathyroid glands [1–5]. In a previous study, Lewin et al. designed an experimental rat model in which an isogenic kidney transplantation normalized the glomerular filtration rate (GFR) of severely uremic rats [6, 7]. They demonstrated that the significantly elevated plasma levels of PTH in uremia became normal within one week of the kidney transplantation. This happened despite persistently suppressed gene expression of *CaR* and *VDR* one week after the transplantation and despite that normalization of these genes did not occur until four weeks after surgery, as illustrated in Figure 1. These results may indicate the existence of other regulatory pathways which are involved in parathyroid CaR and VDR signalling. *In vitro*, another situation occurs—the rapid reduction in the expression of parathyroid *CaR *[3]. The reason for this *in vitro* reduction is not completely understood at the molecular level. The parathyroid cell in culture loses its phenotype and its responsiveness to changes in extracellular calcium, making it challenging to study changes in the expression of different genes in the parathyroids. 

As the parathyroids apparently can normalize the secretion of PTH despite reduced expression of *CaR* and *VDR*, the question arises whether this sustained low expression occurs due to epigenetic events, for example, methylation of cytosine nucleotides in CpG islands [8]. The term “epigenetics” describes changes in gene activity in the absence of a change in DNA sequence [9]. Such methylation may distort the transcription factor-binding sites causing transcriptional silencing [10]. Together with histone modifications, these epigenetic events might be reversible in time and tissue [11–13]. Epigenetic events are routinely found in various forms of cancer in tissues like colon, brain, liver, blood, breast, and lung [14–18], but also in chronic kidney disease [8]. CpG islands have been identified in the *CaR* and *VDR* genes [19]; methylation of these regions has been detected in different neoplasms [20], and low gene expression due to promoter methylation can be restored by 5-deoxy-3′-azacytidine, an inhibitor of DNA methylation [21]. Research demonstrating the importance of the hypermethylation of CaR and VDR in carcinogenesis makes the *CaR* and *VDR* genes interesting candidates for promoter methylation analysis in parathyroid hyperplasia [20, 22].

Further, vitamin D might be linked to epigenetic control of chromatin structure [23–26], since the unresponsiveness of malignant human prostate cells to vitamin D treatment can be reversed by treating the cells with drugs reversing the epigenetic state of the cell (DNA methylation and histone modifications) [25]. 

We launched the present study to examine whether the low expression of parathyroid *CaR *and *VDR* in uremia was associated with changes in methylation levels *in vivo* or *in vitro*. Therefore, we examined the methylation levels of parathyroid *CaR *and *VDR* genes in both normal and uremic rats. Further, we examined normal parathyroid glands *in vitro* and analyzed them for aberrant methylation levels. In these experiments, we performed qPCR to validate the expression of *CaR* and *VDR*, as well as *PTH* and *klotho*, a new hormone of importance in parathyroid physiology [1]. 

The present study found no signs of methylation in the parathyroids of uremic or normal rats, indicating that changes in methylation levels are not involved in the low expression of parathyroid *CaR* or *VDR* genes in uremia, either *in vivo* or *in vitro*.

## 2. Materials and Methods

### 2.1. Ethics Statement

We performed the experimental studies on rats in accordance with the Danish law on animal experiments; the Animal Experiments Inspectorate at the Ministry of Justice, Denmark approved these studies (permit number 2007/561-1278). Every effort was made to minimize suffering.

### 2.2. Bioinformatics

**Figure 2:**
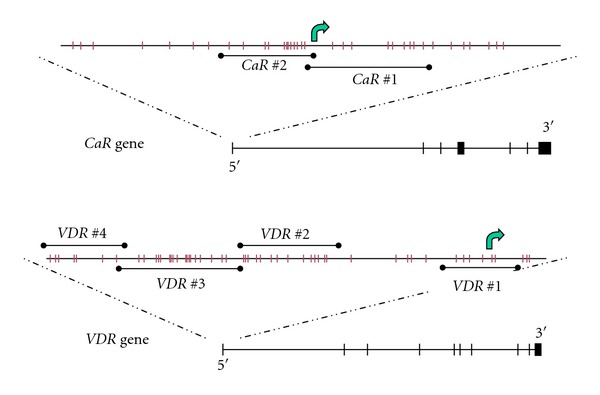
Outline of CpG islands in *CaR* and *VDR* promoters. The solid horizontal lines labeled CaR#1-2 and VDR#1-4 indicate the locations of the PCR products, and the arrow indicates the exon 1 start. The small vertical lines in the promoter regions indicate individual CpG dinucleotides.

We searched for CpG islands in the genes of CaR and VDR using the internet site http://cpgislands.usc.edu/, using default search parameters, as well as http://genome.ucsc.edu/cgi-bin/hgGateway. We designed PCR primers using the software Methyl Primer Express v1.0, selecting primers that target preferably non-CpG-containing areas. The CpG islands analyzed were located from −250 basepair (bp) to +300 bp from exon 1 of the *CaR* gene and from −800 bp to +200 bp from exon 1 of the *VDR *gene (see Figure 2). The analyzed downstream area of VDR near exon 10 is not considered a promoter region, and it only served as a methylation control region to positively demonstrate methylation.

### 2.3. DNA, PCR, and Melting Curve Analysis

We extracted genomic DNA from parathyroid glands using a QIAamp DNA Mini Kit (cat. no. 51304, Qiagen AB, Sollentuna, Sweden). The bisulfite conversion of the DNA, changing cytosine nucleotides to uracil, was performed using the EZ DNA Methylation Kit (cat. no. D5001, Zymo Research, Irvine, CA, USA). Briefly, 500 ng DNA from each parathyroid gland was treated for 16 hours in the dark at 50°C and eluted in 10 *μ*L elution buffer. We used commercial highly methylated genomic rat DNA (cat. no 80-8065-RGHM5) and commercial nonmethylated genomic rat DNA (cat. no. 80-8066-RGUM5, EpigenDx, Worcester, MA, USA) as standards. One *μ*L of bisulfite-treated genomic DNA was used for PCR.  Table 1 lists the PCR primers used. The PCR products were verified by agarose gel to confirm size and a single band. The endpoint PCR temperature profile was 94°C for 10 minutes, 30 cycles of 94°C for 30 seconds, 53°C for 45 seconds, and 72°C for 90 seconds and, finally, a melting curve analysis from 50°C to 94°C. The endpoint PCR kit used was a SYBR Brilliant II (cat. no. 600828, Agilent, CA, USA). The melting curve analysis was based on the fact that the SYBR dye can only bind to double-stranded DNA and not single-stranded DNA. The SYBR dye fluoresces only when bound to the double-stranded DNA. When the PCR products are heated from 50°C to 94°C, the two DNA strands will separate at some specific temperature, the SYBR dye will dissociate from the DNA, and the fluorescent signal will decrease. The melting curve analysis, a built-in feature of the Roche Lightcycler 480 II software, calculates the loss of fluorescence with increasing temperature (from 50°C to 94°C) by measuring the slope of the initial raw fluorescence data.

### 2.4. RNA and qPCR

**Figure 3:**
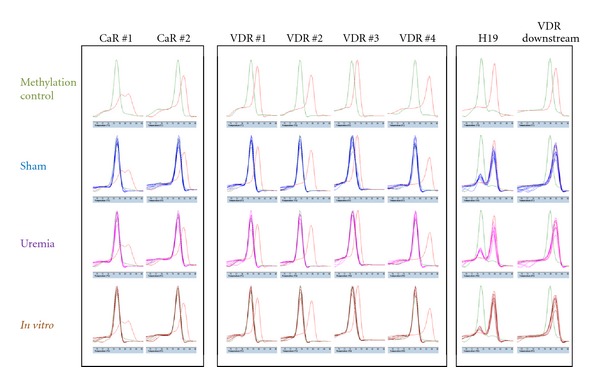
Methylation detection of *CaR* and *VDR* promoter regions in parathyroid glands. Analysis of the *CaR* and *VDR* promoter regions by PCR melting curve analysis after bisulfite treatment of genomic DNA. *CaR* is covered by PCR products *CaR*#1-2, and *VDR* is covered by PCR products *VDR*#1–4. Commercial low- and high-methylated rat DNA is analyzed in all of the PCR reactions shown in the “Methylation control” panel in green and red, respectively; it is also included in the “Sham,” “Uremia,” and “*in vitro*” panels. Control of methylation detection verified high levels of methylation in *H19* and *VDR* downstream gene regions.

**Figure 4:**
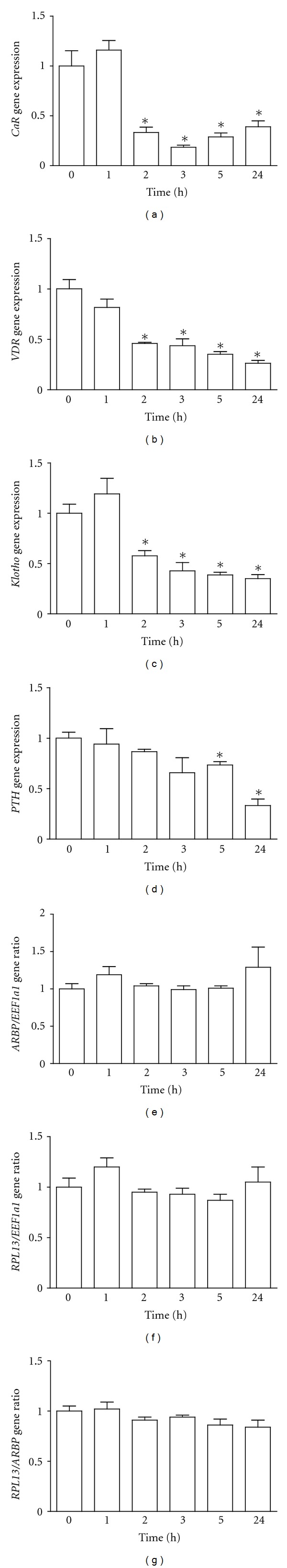
*In vitro* expression of *CaR, VDR, klotho*, and *PTH* in parathyroid tissue cultures over time. (a)–(d): gene expression of *CaR, VDR, klotho*, and *PTH* over time. (e)–(g): gene expression of housekeeping genes *EEF1a1, ARBP*, and *RPL13* over time. (*) Statistical significance of *P* < 0.05 is indicated, when compared to the zero-hour group. Gene activity is shown as gene ratios.

RNA was extracted using a Trizol extraction kit, (cat. no. T9424, Sigma-Aldrich, St. Louis, MO, USA). We performed cDNA synthesis using 200 ng RNA that was reverse transcribed using an Invitrogen cDNA kit (cat. no. 18080-051, Grand Island, NY, USA) with 125 pmol 15-mer random primer at a 50°C synthesis temperature. We used 5 *μ*L of cDNA (diluted ten times) for qPCR [1]. PCR experiments were performed as follows: we used 15 pmol of each primer in a total volume of 20 *μ*L PCR reaction. The temperature profile was 94°C for 10 minutes, 35 cycles of 94°C for 30 seconds, 60°C for 45 seconds, and 72°C for 90 seconds. We calculated *CaR*, *VDR, klotho, *and* PTH* gene expressions, using the second derivative method to calculate the Cp value for each sample against a standard dilution series to incorporate the efficiency of the PCR reaction, and a gene concentration was calculated. We calculated the mean activity of the housekeeping genes* EEF1a1, RPL13*, and *ARBP* for each sample and used the results to calculate the gene activity of *CaR*, *VDR*, *klotho*, and* PTH* (shown in Figures 3(a)–3(d) as gene ratios). In Figures 4(e)–4(g), the gene ratios of one housekeeping gene were calculated using the activity of each of the other housekeeping genes to show healthy, intact parathyroid tissue. 

### 2.5. Animals

Adult male Wistar rats, weighing 225 g (Taconic, Ballerup, Denmark), were kept in a controlled environment with a 12-hour light-dark cycle, a constant temperature (22°C), and a relative humidity of 70 percent. We provided all rats with free access to food and water and fed them either (a) a standard rat chow diet containing 0.9 percent calcium, 0.7 percent phosphorus, and vitamin D (600 IU per kg^−1^) or (b) a high-phosphorus rat chow diet containing 0.9 percent calcium, 1.4 percent phosphorus, and vitamin D (600 IU per kg^−1^).

We divided the rats into the following experimental groups:

group 1: *in vivo*: promoter methylation analysis of *CaR* and *VDR* in the parathyroid glands of uremic versus sham-operated rats;
group 2: *in vitro*: promoter methylation analysis of *CaR* and *VDR* in sham-operated rat parathyroid glands at time points 0 and 24 hours;
group 3: *in vitro*: gene expression of *CaR*, *VDR*, *klotho*, and *PTH* in rat parathyroid glands at time points 0, 1, 2, 3, 5, and 24 hours.

### 2.6. 5/6 Nephrectomy

We performed one-step 5/6 nephrectomy to induce uremia. In order to induce severe s-HPT, we gave a 5/6 nephrectomized group of nine rats a high phosphorus diet. Ten sham rats were given a standard diet. The duration of uremia was eight weeks. On the day of the nephrectomy, the rats received anaesthesia with Hypnorm/midazolam (Panum Institute, Copenhagen, Denmark). Additional doses were given, when required, to maintain a steady level of anaesthesia, and the rats were given carprofen (Rimadyl, 50 mg/mL, Pfizer, Copenhagen, Denmark) subcutaneously at a dose of 30 *μ*L/rat as pain relief for the following three days. We made every effort to minimize suffering.

### 2.7. Parathyroidectomy

After eight weeks of uremia, the parathyroid glands were removed, and the glands were snap frozen in liquid nitrogen for subsequent promoter methylation analysis.  Table 2 shows the plasma parameters of these rats. For the *in vitro* experiments, the parathyroid glands were removed and immediately placed in a 37°C incubation medium.

### 2.8. Culture of Parathyroid Glands *In Vitro*

Parathyroid glands from normal rats were cultured *in vitro* for various time intervals: 0, 1, 2, 3, 5, and 24 hours (*n* = 3 at each time point) and cultured in DMEM-HAM's F12 medium with a calcium concentration of 1.2 mM [27]. The medium was changed after 1, 2, 3, 4, and 23 hours. We assessed gene expression after the incubation by qPCR. 

We performed a second set of experiments in order to assess the methylation status of glands grown for 24 hours *in vitro*. Parathyroids from four rats served as control at time = zero, and we grew the parathyroids from seven rats *in vitro* for 24 hours.

### 2.9. Plasma Measurements

We obtained and analyzed blood samples using a Vitros 150 (Ortho-Clinical Diagnostics, Rochester, NY, USA) for plasma phosphate, urea, and creatinine. We measured plasma ionized calcium (p-Ca^2+^) using an ABL 505 (Radiometer, Copenhagen, Denmark). Rat plasma PTH was measured using a rat bioactive intact PTH ELISA assay from Immutopics (San Clemente, CA, USA).

### 2.10. Statistic Calculations

We used Student's t-test and presented the data as mean ± standard error of the mean (SEM). We set the statistical significance at *P* < 0.05. 

## 3. Results

We examined the CpG islands in the *CaR* and *VDR* genes, from −250 to +300 base pairs (bp) from exon 1 of the *CaR* gene and from −800 to +200 bp from exon 1 of the *VDR *gene, as shown in Figure 2. The analyzed downstream area of *VDR* near exon 10 is not a promoter region and only served as a positive methylation control region. *H19 *served as a second control.

We initially performed experiments to ensure that SYBR melting temperature profiling, previously used to detect changes in methylation levels [28–30] would detect both low and high levels of methylation in PCR products. We included positive and negative methylation standards, which clearly showed distinct changes in melting temperature in each of the PCR reactions analyzed, as shown in the upper row of Figure 3.  Table 1 presents the primers and the number of CpGs in each PCR product that resulted in changes in the melting temperature profile when methylated. 

We validated the bisulfite DNA conversion method in all samples by analyzing the methylation levels of two known methylated gene regions—the imprinted gene *H19* [31] and a downstream area of *VDR* near exon 10 [32], as shown in Figure 3. Two peaks in *H19* were found in all samples, as expected, indicating methylation on one strand and not the other. The present study analyzed, in rats, the highly methylated region downstream of VDR near exon 10 also reported in humans. We found high levels of methylation of both DNA strands in every sample, detecting only one peak, which coincided with the high methylation control sample. Thus, the bisulfite conversion reaction performed well, and temperature melting profiling clearly detected the expected changes in the methylation level in these positive control gene regions.

In order to analyze the methylation levels of the *CaR* and *VDR* genes in our *in vivo* experiments, we performed gene-specific endpoint PCR and analyzed the PCR products by melting temperature analysis, as shown in Figure 3. Every peak coincided with the negative methylation standard; thus, no changes were observed in the melting temperature of any of the PCR products from the sham or uremic parathyroid glands.

We grew tissue cultures of parathyroid glands *in vitro* at various time intervals for up to 24 hours. Figures 4(a)–4(d) outline the gradual reduction of the gene expression of parathyroid *CaR, VDR, klotho*, and *PTH* over time. We found the expressions of *CaR, VDR,* and *klotho* expressions all significantly downregulated at two hours, whereas we first observed a significant downregulation of *PTH* at 24 hours. We performed a second set of experiments to examine whether methylation of CpG islands in *CaR* and *VDR* coincided with the reduction of parathyroid *CaR* and *VDR* expression. We compared freshly harvested parathyroid glands to parathyroid glands grown *in vitro* for 24 hours. We found no aberrant melting curves, indicating that the parathyroid glands in culture had the same low methylation levels at time zero and at 24 hours. Figures 4(e)–4(g) show the stable expression of three housekeeping genes over time, ensuring the viability of the parathyroid glands *in vitro*.

## 4. Discussion

Severe uremia is complicated by secondary hyperparathyroidism with hyperplasia of the parathyroid glands and is characterized by low parathyroid gene expression of *CaR* and *VDR* [2, 4, 5, 33, 34]. As s-HPT gets worse, the glands become unresponsive to vitamin D and calcium therapy, indicating that the parathyroid cells with low expression of *CaR* and/or *VDR* can no longer convey calcium or vitamin D signals. Lewin et al. showed in 2002 that plasma PTH, together with plasma Ca^2+^, phosphate, creatinine, and urea, normalized after an experimental isogenic kidney transplantation in rats, despite persistently low parathyroid expression of *CaR* and *VDR*; only several weeks later did the expression of* CaR* and *VDR* become normal in the parathyroids [2, 35, 36]. The delay in the restored expression of *CaR* and *VDR* in this model of reversal of uremia by Lewin et al. stresses the importance of a search for not-yet-identified mechanisms that might control *CaR* and *VDR* genes in the parathyroids. 

The present study examined the methylation status of the *CaR* and *VDR* promoter regions. We found no indication of methylation, as the results of all samples coincided with the negative methylation standard, as shown in Figure 3. 

No parathyroid cell line exists for culturing, leaving researchers freshly harvested parathyroid tissue/cells to examine. However, parathyroid glands grown *in vitro* present a significantly reduced expression of the *CaR* gene within the first 24 hours of culture [3, 34]. In the present study, we assessed the expressions of key parathyroid genes: *CaR, VDR, PTH,* and *klotho*. As expected, we observed a significant decline of the expression of *CaR* within two hours of incubation, reaching a lower steady level after 5 to 24 hours, as shown in Figure 4(a). Similarly, the expression of *VDR* and *klotho* genes also declined after two hours of incubation, reaching a lower steady level after 5 to 24 hours, as shown in Figures 4(b)-4(c). The expression of the *PTH* gene also declined—but slowly, over time—reaching a nadir at 24 hours, as shown in Figure 4(d). In contrast, the expression of the three housekeeping genes was stable during the 0 to 24 hours of culture, as shown in Figures 4(e)–4(g), suggesting the persistent viability of the parathyroid cells.

We studied the methylation levels of the *CaR* and *VDR* promoters *in vitro* at time zero and after 24 hours in order to examine their association to the low expression of these genes. As shown in Figure 3, we detected no changes in methylation levels over time, indicating that the low expression of parathyroid *CaR* and *VDR* genes *in vitro* was not associated with methylation.

The *CaR* gene is expressed not only in the tissues, where it is primarily involved in calcium homeostasis, such as the parathyroid glands, the C-cells of the thyroid gland, the kidney, and bone, but also in a number of other tissues, where it is implicated in the regulation of multiple cellular functions. It has been proposed that CaR plays an important role in the regulation of intestinal cell proliferation and differentiation. Stimulation of *CaR* expression in colon epithelial cells was shown to induce an inhibition of proliferation [37, 38]. The loss of expression of *CaR* was associated with poor differentiation and malignant progression [20, 39]. Recently, epigenetic inactivation of *CaR* expression by promoter hypermethylation was demonstrated in colorectal carcinogenesis [20].

Activation of CaR is related to the regulation of parathyroid cell proliferation. This was proven indirectly by the observation that the administration of CaR agonists led to the inhibition of parathyroid cell proliferation in uremic rats [40]. However, as our study demonstrates the loss of expression of *CaR* in parathyroid hyperplasia secondary to uremia is, unlike colonic neoplasms, not associated with hypermethylation of the *CaR* gene promoter.

*VDR* has been demonstrated in a broad range of tumors and malignant cell types, and the inhibition of cancer cell growth, angiogenesis, and metastasis by calcitriol has been shown. For colon and breast cancer cells, an inverse relationship between *VDR* levels and degree of differentiation has been described [41–43]. Recent research has shown the hypermethylation of the *VDR* gene promoter region in primary breast tumors and its absence in normal breast tissue [44], and the role of the epigenetic silencing of *VDR* by promoter hypermethylation as the mechanism behind the resistance of breast cancer cells to calcitriol has been proposed. The present results do not, however, support a similar mechanism in parathyroid hyperplasia in uremia. It should, however, be emphasized that experimental secondary hyperparathyroidism does not fully resemble the advanced hyperparathyroidism with clonal transformation which is seen in humans, and it can therefore not be ruled out that CaR or VDR promoter methylation might exist in the human setting.

Some limitations of the present investigation should be stressed. CpG island methylation often goes hand in hand with histone modifications [45–50]. In the present study, we focused only on the methylation status and did not assess the histone modification profile in the *CaR* and *VDR* gene areas; thus, histone modifications may still play a role in the delayed *CaR* and *VDR* expression profile in the parathyroids. Furthermore, the present results do not exclude epigenetic mechanisms in the upstream signalling pathways that regulate *CaR* and *VDR* gene expression or in other areas of the *CaR* or *VDR* gene regions. The method used to detect changes in methylation levels can only detect overall changes and will not reveal if one locus becomes methylated in combination with loss of methylation at another locus in the PCR product. 

## 5. Conclusion

In uremia, severe hyperparathyroidism is characterized by low parathyroid expression of *CaR* and *VDR*. Disturbances in *CaR *and* VDR* gene methylation patterns have been shown in tissues with rapid growth, such as in various cancer tissues, where these epigenetic changes were responsible for the uncontrolled cell growth. Therefore, we examined the parathyroid glands from uremic rats for changes in the methylation levels of the *CaR* and *VDR* genes. We performed the methylation analysis of the CpG islands in the *CaR *and *VDR* genes to examine whether uremic parathyroid glands exhibited epigenetic changes. We found no overall changes in the melting temperature curves of any of the PCR products, which we analyzed in this rat model of uremic s-HPT and in rat parathyroid tissue *in vitro*. We concluded that methylation is not associated with the distorted gene expression of *CaR* and *VDR* in experimental uremic secondary hyperparathyroidism or in parathyroid glands grown *in vitro*. 

## Figures and Tables

**Table 1:** PCR primers used for methylation detection by melting temperature profiling.

Name	Location^#^	5′-3′ sequence	Size^$^	Number of CpG
Promoter				
CaR #1F	(−172)	AGTTTGGGAATGGTTAGAGTTTTAT	171 bp	12
CaR #1R	(−2)	ACTCCCTAAATCTCTCAAATCAAC		
CaR #2F	(−28)	AAGGTTGATTTGAGAGATTTAGG	185 bp	9
CaR #2R	(+157)	CTACTACCTCCCCrCAAACCCTCT		
VDR #1F	(−58)	TTTTTGTTTGTTAAAAGGTTGTTGT	125 bp	6
VDR #1R	(+67)	AATCTAATCACTCACCCAAAACTC		
VDR #2F	(−374)	GTTTATAGTAGATTGGGTAGAATTA	175 bp	12
VDR #2R	(−200)	CCTACCTTATAAAAAACTCTAAAATC		
VDR #3F	(−576)	GTTTTTTTGAGTTAATTTTAATTAGTGG	169 bp	7
VDR #3R	(−389)	TAATTCTACCCAATCTACTATAAAC		
VDR #4F	(−678)	AGGATTTGGGAGGTAGAGATTTTAG	188 bp	18
VDR #4R	(−510)	CCACTAATTAAAATTAACTCAAAAAAAC		

Control				
H19 F	(−6233)	GAGGGTAGGATATATGTATTTTTAGGTTG	185 bp	13
H19 R	(−6049)	AAAAAAATTCAATCTCAATTACAATCTATTT		
VDR DS F	(+48639)	AAGGGGTGTGAGTTTTATGTTA	269 bp	12
VDR DS R	(+48908)	AAACAATAAACATCCTATCTCCC		

^#^Relative location of the 5′ nucleotide according to exon 1.

^$^Bp denotes DNA base pair.

**Table 2:** Plasma parameters of the sham and uremic group of rats.

	PTH pg/ml	Ca^2+^ mM	P mM	Urea mM	Creatinine *μ*M
Sham, *n* = 10	90 ± 39	1.29 ± 0.01	1.36 ± 0.06	6.6 ± 0.5	39 ± 1.4
Uremia, *n* = 9	1851 ± 343	1.14 ± 0.06	2.40 ± 0.26	17.6 ± 1.5	94 ± 12
